# Discovery selective acetylcholinesterase inhibitors to control *Tetranychus urticae* (Acari: Tetranychidae)

**DOI:** 10.1093/jisesa/iead073

**Published:** 2023-08-14

**Authors:** Jiachen Wang, Yang Cao, Bin Lai, Yongshuai Liu, Chao Li, Chunya Bu

**Affiliations:** Key Laboratory of Northern Urban Agriculture of Ministry of Agriculture and Rural Affairs, College of Bioscience and Resource Environment, Beijing University of Agriculture, Beijing 102206, China; Center for Growth, Metabolism and Aging, Key Laboratory of Bio-Resources and Eco-Environment of Ministry of Education, College of Life Sciences, Sichuan University, Chengdu 610064, China; Key Laboratory of Northern Urban Agriculture of Ministry of Agriculture and Rural Affairs, College of Bioscience and Resource Environment, Beijing University of Agriculture, Beijing 102206, China; Key Laboratory of Northern Urban Agriculture of Ministry of Agriculture and Rural Affairs, College of Bioscience and Resource Environment, Beijing University of Agriculture, Beijing 102206, China; Key Laboratory of Northern Urban Agriculture of Ministry of Agriculture and Rural Affairs, College of Bioscience and Resource Environment, Beijing University of Agriculture, Beijing 102206, China; Key Laboratory of Northern Urban Agriculture of Ministry of Agriculture and Rural Affairs, College of Bioscience and Resource Environment, Beijing University of Agriculture, Beijing 102206, China

**Keywords:** anticholinesterase, *Tetranychus urticae*, human, selective inhibitor, mite control

## Abstract

The two-spotted spider mite, *Tetranychus urticae* Koch, has a broad host plant range and presents an extreme capacity for developing pesticide resistance, becoming a major economic pest in agriculture. Anticholinesterase insecticides still account for a big part of global insecticide sales. However, there is a growing concern about the serious resistance problems of anticholinesterase insecticides and their nontarget toxicity. In this study, structure-based virtual screening was performed to discover selective AChE inhibitors from the ChemBridge database, and 39 potential species-specific AChE inhibitor were obtained targeting *T*. *urticae* AChE, but not human AChE. Among them, compound No. 8 inhibited AChE from *T*. *urticae*, but not from human, and had an inhibitory activity comparable to that of eserine. Compound No. 8 had dose-dependent toxicity to *T*. *urticae* in glass slide-dipping assay and had significant mite control effects in a pot experiment, but required a high concentration to achieve similar control effects to spirodiclofen. The toxicity evaluation suggested that compound No. 8 had no acute toxicity on pollinator honey bees and natural predator *N. californicus* and did not affect strawberry growth in our assay. Compound No. 8 is a potential lead compound for developing novel acaricides with reduced nontarget toxicity.

## Introduction

The two-spotted spider mite, *Tetranychus urticae* Koch (Acari, Tetranychidae), is an economically important and extremely polyphagous herbivorous pest, feeding on more than 1,100 plant species that belong to over 140 agricultural crops (Grbić et al. 2011, Dermauw et al. 2020). The mites are sap-suckers and cause crop loss in severe infestations. The detrimental effects of spider mites on agriculture might markedly increase with intensifying global warming, as they develop rapidly at high temperatures (Grbić et al. 2011). Mite control depends largely on using chemical pesticides. Spider mites have developed resistance to most of the currently used acaricides and have currently been reported as the most resistant pest worldwide (Grbić et al. 2011, Adesanya et al. 2020). Rapid development, high fecundity, and haplodiploid sex determination may contribute to their rapid evolution of insecticide resistance (Grbić et al. 2011, Van Leeuwen and Dermauw 2016). Some insecticides, such as organophosphate and carbamate, are known to be probably toxic to mammals and nontarget species, such as natural predators and pollinators, which has become a growing concern (Lang et al. 2012). The decrease in predatory mites and honeybees may limit crop yields. Research into highly effective insecticides with low nontarget toxicity is urgently needed.

Insecticides that target acetylcholinesterase (AChE; EC 3.1.1.7) still account for quite a big part of the global insecticide sales (Lang et al. 2012). In the past decades, a series of AChE crystal structures and their inhibitor complex were elucidated, shedding light on the molecular mechanism of action of the insecticides and resistance mutations (Lang et al. 2012, Hadiatullah et al. 2022). The AChEs from different species and the various *aces* gene types are poorly conserved (Lang et al. 2012). Attempts have been made to develop new insecticides with reduced off-target toxicity and low propensity for insect resistance (Pang et al. 2012). Some AChE1 active site structural variations were previously identified, when comparing *T*. *cinnabarinus* and human AChE (Bu et al. 2015b). AChEs from different species respond differently to inhibitors (Lang et al. 2012). Selective AChE inhibitors designed to control mites can therefore be developed to spare nontarget species (Lang et al. 2012).

In this study, the structures of AChE from *T. urticae* and from humans were compared, particularly the structures of the catalytic pocket. Structure-based virtual screening was performed to discover potentially selective AChE inhibitors from the ChemBridge. These compounds were then tested for their inhibitory activity on human and mite AChE. The potential of the compounds to fight *T*. *urticae* infestations was further analyzed.

## Materials and Methods

### Mites

Colonies of a pesticide-susceptible strain of *T. urticae* (red form) are grown under insecticide-free conditions on cowpea leaves at 25 ± 2 °C, 45 ± 5% RH, and a photoperiod of 16L:8D h in the growth chamber. These mites were assayed each year for high sensitivity to pesticides using the glass slide-dipping method according to FAO standards (Bu et al. 2015a).

A wild-type strain of *T. urticae* (red form) under normal pesticide pressure was collected from the peach orchard in Pinggu District, Beijing, China.

### Molecular Docking

The amino acid sequence of the AChE of *T. urticae* (red form) was retrieved from GenBank (accession number: AGI96546.1). Protein modeling of *T. urticae* (red form) AChE was constructed as previously described on the I-TASSER server (https://zhanglab.ccmb.med.umich.edu/I-TASSER/) (Bu et al. 2015b). Structural templates (human AChE PDB: 4qww, 4bdt, 1qo9, 4m0e, 4tpk, 2xb6, 5x61) were first identified from the PDB by a multiple threading approach (Zheng et al. 2019), and then the overall structure was obtained by template-based fragment assembly simulations (Yang and Zhang 2015), followed by structure optimization using a side-chain packing software CISRR to further improve the side-chain prediction quality and eliminate the atomic clashes (Zhang 2008, Cao et al. 2011, Cao and Li 2014).

For virtual screening, the ChemBridge database was used. The 3D structures of the compounds were generated using OpenBabel (O’Boyle et al. 2011). Molecular flexibility analysis was performed to eliminate extremely flexible molecules from the database, followed by docking to the mite AChE catalytic site. After performing rigid body docking with default parameters using DOCK 6.0, the compounds were ranked according to their affinity for AChE (Allen et al. 2015). The top 10,000 compounds were recomputed for the second-round docking by a ligand-flexible docking program AutoDock Vina, using the exhaustive parameter of 12 for a relatively more exhaustive conformational search (Trott and Olson 2010). The structures with the highest affinity for mite AChE were selected.

The docking of these 10,000 compounds with the human AChE structure (PDB: 1B41) was performed as above. Those compounds that had a high affinity for the mite AChE but a low affinity for the human AChE were used in the enzyme activity assays that followed.

### AChE Inhibition Assay

AChE activity was determined by a modified Ellman’s method (Anazawa et al. 2003, Ilg et al. 2011, Bu et al. 2015b). Preincubated with different compounds for 20 min at 4 °C and 10 min at room temperature, the purified AChE proteins or mite extracts were thoroughly centrifuged to remove the precipitate, followed by the transfer of each aliquot to the reaction wells. The remaining AChE activities were measured by adding 0.3 mM 5,5ʹ-dithiobis-(2-nitrobenzoic acid) (DTNB, Sigma–Aldrich) and 0.3 mM acetylthiocholine iodide (ATChI, Sigma–Aldrich) at 30 °C and 412 nm over 5 min using a BIO-RAD X-Mark microplate reader. On each plate, 4 replicates were used for each treatment. Negative (solvent), positive (no inhibitor), and eserine inhibitor controls were included in each plate. The percentage activity of AChE enzyme treated with the test compound was calculated using the formula: (*T* − *N*)/(*P* − *N*) ×100%, where *T* is test compound absorbance, *N* is negative control absorbance, and *P* is positive control absorbance. Inhibition values were obtained from 3 independent experiments. Dose–response curves were constructed using inhibitor concentrations ranging from 0.1 to 5 mM, and IC_50_ values were obtained as previously described (SPSS 13.0, SPSS Institute 2004) (Bu et al. 2015b).

Mite extracts were prepared as follows (Anazawa et al. 2003). Mites were thoroughly crushed with liquid nitrogen, suspended in cold 50 mM Tris–HCl (pH 8.0) containing 0.1% Triton X-100, and centrifuged to remove the precipitate.

The purified recombinant mite AChE protein was prepared as follows (Kwon et al. 2012, Bu et al. 2015b). The *ace* coding region was PCR amplified, inserted into pET-30a, and expressed in *E. coli* BL21(DE3) cells as described previously (Bu et al. 2015b). Cell extracts were collected 7 h after induction by 1 mM IPTG at 28 °C, and the expression of AChE protein was verified by western blotting using rabbit anti-mite AChE antibodies (Bu et al. 2015b). The recombinant mite AChE protein was purified according to the instructions for the Ni-NTA Superflow column (Amersham Pharmacia Biotech, Little Chalfont, UK). Amicon Ultra-15 was used to concentrate the AChE protein. SDS–PAGE and western blotting were used to determine the purity and specificity of the AChE protein obtained. Protein concentrations were determined using a BCA protein assay kit (Thermo Fisher Scientific, Waltham, MA, USA). The purified recombinant AChE protein of *T*. *urticae* was used in assays at a final concentration of 0.5 μM.

Human red blood cell extracts were prepared as described previously (Bu et al. 2015b). Human erythrocytes were rinsed with 50 mM Tris–HCl containing 1 mM EDTA (pH 7.4) and normal saline, followed by 8–10 cycles of repeated centrifugation and washing with 10 volumes of 10 mM Tris–HCl (pH 7.4). The membranes were then sonicated for 5–10 s in 50 mM Tris–HCl (pH 7.4) containing 0.1% Triton-X 100 (Pang et al. 2009). The pellet was removed by centrifugation, and the supernatants were diluted 50-fold in 50 mM Tris–HCl (pH 7.4) containing 0.1% Triton X-100 for a later assay.

### Measurement of Contact Toxicity of Active Compounds Against *T. urticae*

The contact toxicity of various compounds on *T. urticae* was determined using the glass slide-dipping method according to FAO (Bu et al. 2015b). The compounds were diluted in sterile distilled water containing 1% Tween 80 (Sigma–Aldrich, St. Louis, MO, USA) and 0.6% DMSO (Sigma–Aldrich, St. Louis, MO, USA). Negative control was the solvent. Double-sided tape was used to attach female adult mites to one end of a glass slide (10 × 2 cm). After the mites were immersed in drug solutions for 5 s, the excess solution was absorbed with filter paper and then the mites were kept in an incubator at 25 ± 2 °C, relative humidity 45 ± 5%, and a photoperiod of 16:8 h (light:dark). Mortality of mites was checked after 24, 48, and 72 h. Mites that did not move when lightly brushed were considered dead. The assay was replicated 3 times. LC_50_ values were obtained as previously described (SPSS 13.0, SPSS Institute 2004) (Bu et al. 2015b).

### Effects of Compound No. 8 on Mite Control and Strawberry Growth

The solution containing 1% Tween 80 and 0.6% DMSO was used to dissolve the compound No. 8. The solvent was used as the negative control, and spirodiclofen was the positive control. According to the field layout (Supplementary Table S2), the spraying trials were conducted on 96 commercial strawberry seedlings (*Fragaria* × *ananassa* ‘Red Face’) grown in a chamber (25 ± 3 °C, 65 ± 10% RH) using commercial soil substrate (soil:peat:sand mixture 3:2:1), which was sterilized at 100 °C for 2 h and left to cool before use. Zero, 15, and 30 mites were inoculated on each seedling for control, low inoculation, and high inoculation level group, respectively. A serial concentration of compound No. 8 (0–5 mg/ml) and spirodiclofen (0–0.24 mg/ml) was sprayed on seedlings at 0 day as shown in Supplementary Table S2. Each treatment contained 4 seedlings and was repeated 3 times. The number of live mites was recorded on a daily basis up to 9 days after the treatment.

To check the effects of the compound No. 8 treatments on strawberry growth, chlorophyl content was recorded at 3 random positions of a fully developed leaf in each seedling using a chlorophyl meter (SPAD-502-Minlota Co. Ltd., Japan) at ninth day post-treatment (El-Bialy et al. 2023). Meanwhile, the aerial dry weight was determined after drying at a temperature of 50 °C for a period of 72 h.

### The Toxicity of Compound No. 8 on Honey Bees

All honey bees were collected from the apiary of the Institute of Apicultural Research (40°01ʹ23″N, 116°21ʹ24″E) of Chinese Academy of Agricultural Sciences. The colonies were *Apis mellifera*, housed in standard Langstroth-style equipment, and managed per common best management practices for the region (Dai et al. 2017, 2018). Emerging adults were collected and reared in hoarding cages fed with pollen and 50% sugar water solution (w/v). The acute toxicity of compound No. 8 to honeybees was evaluated including the following treatments: compound No. 8 (0, 0.2, 0.4, 0.8, 1.6, 3.2 mg/l) in sugar water solution, sugar water solution control, and solvent control. There were 4 replicates for each treatment, with 20 adults per treatment from a single colony, while different replicates were taken from a different colony. Bee mortality was determined after 24, 48, and 72 h.

### The Toxicity of Compound No. 8 to *N. californicus*

The commercial colony of predatory mites (*Neoseiulus californicus*) was kept in a climatic chamber, which were maintained in plastic trays and fed 3 times weekly. For each feeding, 3 mite-infested bean leaves were supplied for each tray. The acute toxicity of compound No. 8 to the predatory mites *N. californicus* was evaluated using a sprayer with a serial concentration (0, 0.4, 0.8, 1.6, 3.2 mg/l), and solvent control was used (Ochiai et al. 2007). At 24, 48, and 72 h post-treatment, predatory mite mortality was measured. The experiment was repeated 3 times.

### Statistical Analysis

Graphs were generated using GraphPad Prism version 8.02. One-way ANOVA with Tukey’s honestly significant difference test was used to compare inhibitory activity, mortality, aerial dry weight, and chlorophyl content between the treatments and controls using SPSS 13.0. All the results with a *P*-value < 0.05 were considered statistically significant.

## Results

### Identification of Selective AChE Inhibitor Candidates by Virtual Screening

The AChE structure of *T. urticae* was constructed and then compared with human AChE as previously reported (Li et al. 2021). The AChE structures were constructed with all root-mean-square variations below 2 Å. The overall folding and the deep active site ravine of the catalytic pocket of spider mite and human AChE are very similar. The catalytic triad of *T. urticae* AChE, located at the bottom of the binding region, is highly conserved, indicating its significance. Though AChE structures are highly conserved, significant differences were identified at the catalytic pocket, especially at the peripheral anionic site, located at the entrance of the catalytic pocket between *T*. *urticae* AChE (V105, W156, E316, H369) and human AChE (Y72, Y124, W286, G342), which changed the surface shape of the catalytic pocket of *T*. *urticae* AChE. These structural differences between spider mite and human AChE suggest different catalytic properties and justify research into targets for selective AChE inhibition.

The potential target sites at or near the catalytic pocket of *T*. *urticae* AChE were used to screen AChE inhibitors from Chembridge. The Dock 6 software was used for the primary screening of potential molecules for binding to *T. urticae* AChE. AutoDock Vina was then used to rescore the optimal structures. In total, 100 molecules were selected that were highly affinitive for *T. urticae* AChE but less affinitive for human AChE. Thirty-nine of 100 molecules were selected as inhibitor candidates on the basis of their molecular diversity, shape complementarity, and potential for interaction with the catalytic pocket of *T. urticae* AChE. Samples of 39 compounds were purchased from TopScience for further testing (Supplementary Table S1).

### Inhibitory Activities of Candidate Compounds on *T. urticae* AChE

The 39 compounds obtained from the virtual screening were tested for their ability to inhibit *T. urticae* AChE. The inhibitory activities of 39 compounds were evaluated by measuring residual *T. urticae* AChE activity after exposure. Four of the 39 candidates (compounds No. 8, 9, 16, and 22) were capable of significantly inhibiting *T. urticae* AChE activity (Table 1). The IC_50_ values of the 4 candidates were further determined. Compounds No. 8, 9, 16, and 22 showed concentration-dependent inhibitory activity, and all of them had IC_50_ values comparable to that of eserine (positive inhibitor control).

**Table 1.** Inhibition potency of the compounds against *T. urticae* AChE

Compound	Regressive equation	Correlation coefficient (*R*^2^)	IC_50_ (mM)	95%
8	*y* = 0.21*x* + 0.16	0.99	1.51	0.77–6.07
9	*y* = 0.2763*x* + 0.201	0.90	1.05	0.39–13.16
16	*y* = 0.1259*x* + 0.0366	0.85	2.85	1.638–8.52
22	*y* = 0.5715*x* + 0.1395	0.98	0.63	0.43–0.90

IC_50_ value of eserine (positive control) to *T. urticae* is 1.05 mg/ml.

### Contact Toxicity of Candidate Compounds on *T. urticae*

The contact toxicity of the 4 AChE inhibitors (compounds No. 8, 9, 16, and 22) to *T. urticae* was evaluated using the standard glass slide immersion method. All 4 compounds exhibited concentration-dependent toxicity to *T. urticae* (Table 2). Particularly, compounds No. 8 and 9 had relatively high toxicity to *T. urticae*. However, the toxicity of compounds No. 8 and 9 to *T. urticae* is still lower than that of spirodiclofen (*F*_2, 6_ = 12.120, *P* = 0.004; *F*_2, 6_ = 12.120, *P* = 0.008).

**Table 2.** Contact toxicity of the compounds against *T. urticae* AChE using the glass slide-dipping method

Compound	Treated time (h)	Regressive equation	Correlation coefficient (*R*^2^)	LC_50_ (mg/ml)	95%
8	72	*Y* = 4.17 + 1.96*x*	0.94	2.64	1.68–12.81
9	72	*Y* = 4.19 + 2.22*x*	0.93	2.31	1.71–3.10
16	72	*Y* = 4.35 + 0.63*x*	0.93	10.10	0.53–190.38
22	72	*Y* = 4.01 + 1.37*x*	0.94	5.19	2.03–13.24

### Inhibition Potency of Candidate Compounds at *T. urticae* AChE and Human AChE

**Fig. 1.**
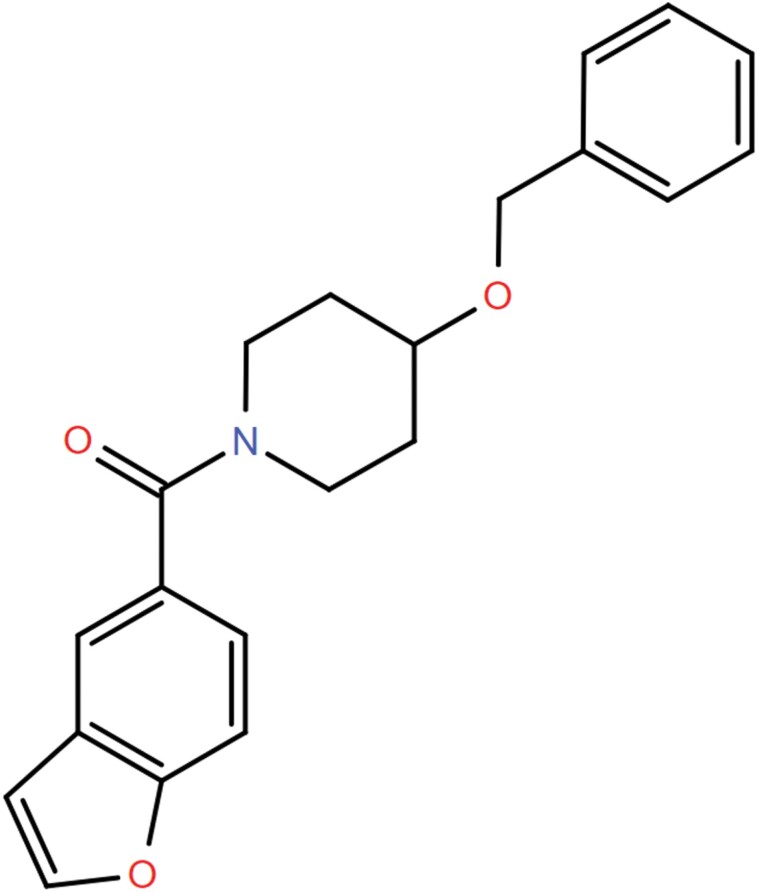
The chemical structure of the compound No. 8 (1-(1-benzofuran-5-ylcarbonyl)-4-(benzyloxy) piperidine).

The inhibition potency of candidate compounds No. 8, 9, 16, and 22 at *T. urticae* AChE and human AChE was determined. Compounds No. 9, 16, and 22 were more effective against human AChE than *T. urticae* AChE (Table 3). However, compound No. 8 was highly selective for *T. urticae* AChE with no observed activity against human AChE and had similar inhibitory activity as that of eserine. The chemical structures of the compound No. 8 are provided in Fig. 1. The compound No. 8 has a piperidine moiety.

**Table 3.** Inhibitory activity of the compounds against *T. urticae* AChE (*Tu*AChE) and human AChE (*h*AChE)^a^

Compound	IC_50_(mM)		Selectivity^b^
	*Tu*AChE	*h*AChE	
8	1.508	—^c^	>100
9	1.05	0.07	0.067
16	2.845	0.48	0.169
22	0.633	0.56	0.88

^a^IC_50_ value of eserine (positive control) to *T. urticae* is 1.05 mg/ml.

^b^Defined as (*h*AChE IC_50_)/(*Tu*AChE IC_50_).

^c^Assayed at the highest concentration.

### Effects of Compound No. 8 on Mite Control and Strawberry Growth

**Fig. 2.**
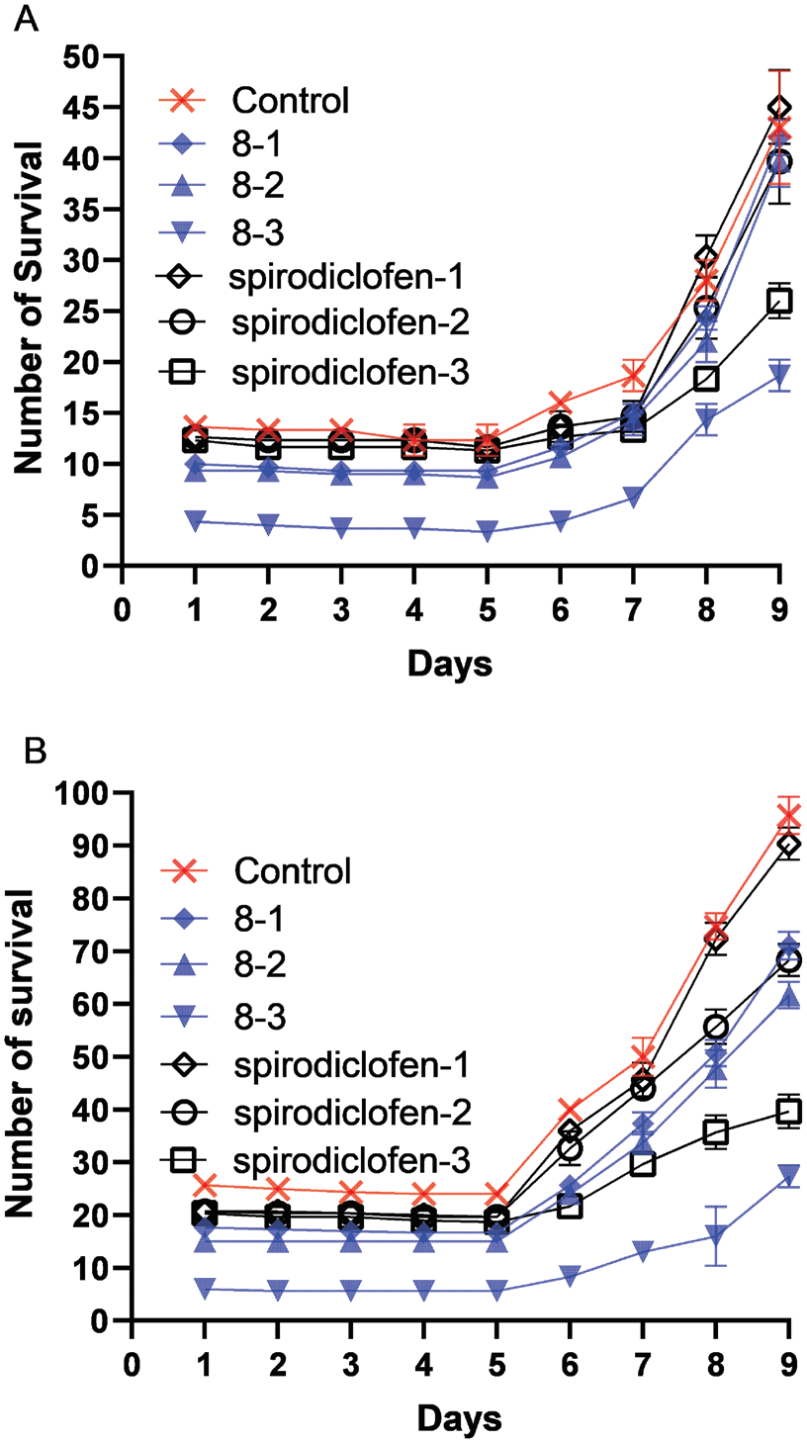
Population trend of the compound-treated group of *T. urticae* in pot experiment. Error bars show SE. 8-1, 8-2, and 8-3 indicate 1, 3, and 5 mg/ml compound No. 8-treated group, respectively. Spirodiclofen-1, spirodiclofen-2, and spirodiclofen-3 indicate 0.06, 0.12, and 0.24 mg/ml spirodiclofen-treated group, respectively. A) Low inoculation level; B) high inoculation level.

The spider mites were inoculated on strawberry seedlings at low and high inoculation levels. The control effects of compound No. 8 on mites were tested using spirodiclofen as a positive control and solvent as a negative control. During the 9 days after compound treatment, high concentration of compound No. 8 was needed to obtain good control effects on *T. urticae* as those of spirodiclofen, and its effects were concentration related (Fig. 2). At 5 mg/ml, compound No. 8 had a good control on pest mite population at both low and high inoculation levels in our assay, compared with the control group (*F*_6, 14_ = 27.298, *P* = 0.000; *F*_6, 14_ = 219.542, *P* = 0.000).

A wild-type strain of *T. urticae* (red form) under normal pesticide pressure was collected from the peach orchard in Pinggu District, Beijing, China. The contact toxicity of compound No. 8 to the wild-type strain *T. urticae* was determined to have a LC_50_ value of 3.41 mg/ml.

**Fig. 3.**
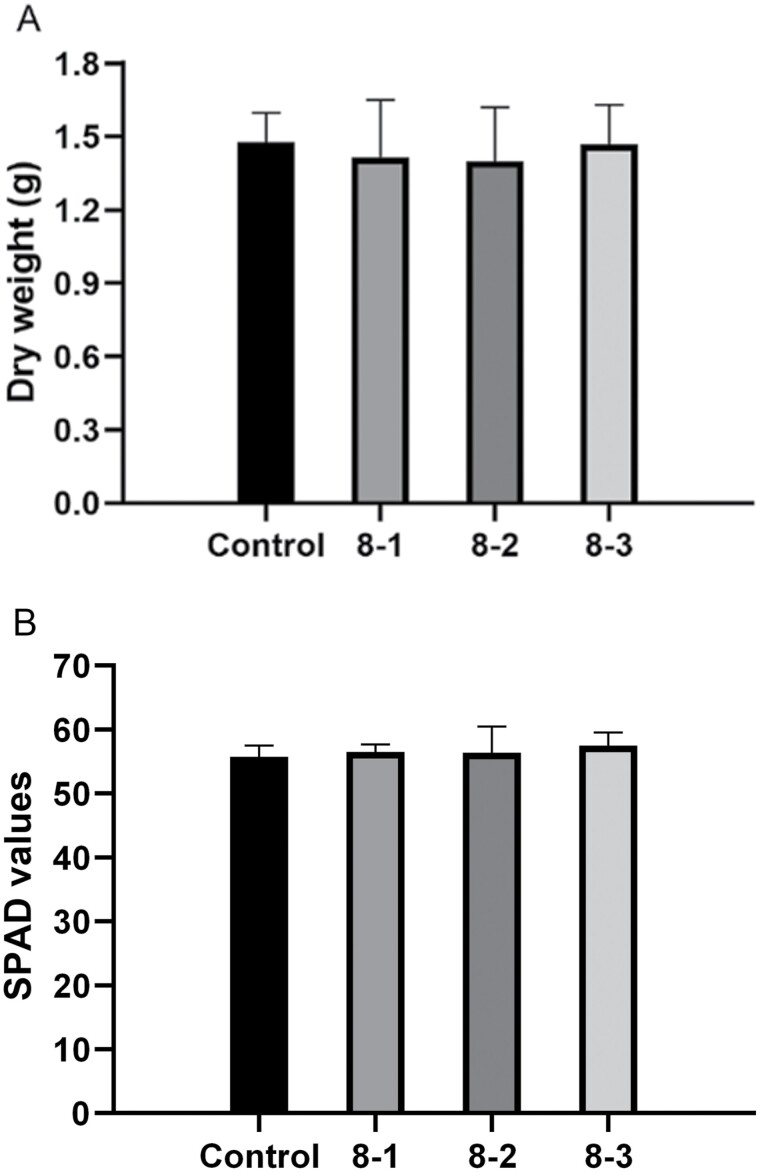
The effects of the compound No. 8 treatments on strawberry seedlings growth. A) Aerial dry weight of strawberry seedlings at ninth day post-treatment; B) chlorophyl content was recorded at 3 random positions of a leaf in each seedling at ninth day post-treatment. Error bars show SE. 8-1, 8-2, and 8-3 indicate 1, 3, and 5 mg/ml compound No. 8-treated group, respectively. There are no significant differences in aerial dry weight and chlorophyl content between compound-treated group and control group, and the data of all groups are measured at the ninth day.

The effects of compound No. 8 on the growth of strawberry seedlings were determined at ninth day after compound No. 8 treatment (Fig. 3). No significant difference was found between treatment with different concentrations of compound No. 8 and the negative control for both aerial dry weight (*F*_3, 8_ = 0.533, *P* = 0.672) and chlorophyl content (*F*_3, 8_ = 0.246, *P* = 0.862). Thus, spraying of compound No. 8 had no effects on the growth of strawberries in our assay.

### Toxicity of Compound No. 8 to Honey Bee and *N. californicus*

The toxicity of compound No. 8 to pollinator honey bees and predatory mites *N. californicus* was further determined. Compound No. 8 was found to have no significant acute toxicity on honey bees and *N. californicus* over the highest concentration that can be determined (Table 4). Thus, compound No. 8 is safe for honey bees and *N. californicus* in our experiments.

**Table 4.** The toxicity of compound No. 8 to *T. urticae*, *A. mellifera* L., and *N. californicus (McGregor)* AChE

Compound No.	LC_50_ (mg/ml)		
	*Tu*AChE	*Am*AChE	*Nc*AChE
8	2.99	—^a^	—^a^

^a^Assayed at the highest concentration.

^b^The LC_50_ value of spirodiclofen (positive control) against *T. urticae* is 0.335 mg/ml.

## Discussion

*Tetranychus urticae* has a broad host plant range and presents an extreme capacity for developing pesticide resistance, becoming one of the most damaging pest mites in agriculture and forestry (Grbić et al. 2011). In addition to insecticide resistance, toxicity of insecticides to nontarget species, such as honey bees and predatory mites, has been reported (Lang et al. 2012, Samaras et al. 2023). There is an increasing demand for new pesticides with novel modes of action against mites. Pesticide application, such as anticholinesterase insecticides, remains the common method of controlling mites (Lang et al. 2012). The fine structures of binding pockets among different AChEs are not highly conserved (Lang et al. 2012). Recently, an increasing number of selective AChE inhibitors with greater activity against insect AChE than human AChE were developed (Pang et al. 2012); however, mites AChE lack the corresponding target site. In this study, we screened novel selective AChE inhibitors for mite control that did not significantly affect human AChE from the ChemBridge database using structure-based virtual screening.

Here, the AChE structures from *T. urticae* and humans were compared. Though AChE structures are highly conserved, significant differences were identified at the catalytic pocket, especially at the peripheral anionic site, located at the entrance of the catalytic pocket between *T*. *urticae* AChE (V_105_, W_156_, E_316_, H_369_) and human AChE (Y_72_, Y_124_, W_286_, G_342_), which changed the surface shape of the catalytic pocket of *T*. *urticae* AChE (Bu et al. 2015b). The spider mite E_316_, H_369_, and V_105_ active sites are important for AChE function, and the discovery of conserved mite-specific residues in *Tetranychus* will enable the development of safer, effective pesticides that target residues present only in mite AChEs, potentially offering effective control against this important agricultural pest (Li et al. 2021). The crystal structures of the SPRY2 domain and Repeat34 domain of diamondback moth ryanodine receptor reveal alternative insect-specific sites, which could be targeted for the development of pest-specific insecticides (Hadiatullah et al. 2022). Based on the structural differences between *T. urticae* AChE and human AChE, selective AChE inhibitors less active against human AChE could be screened.

The structure-based screening is a powerful technique to identify the candidate insecticidal compounds (Liu et al. 2022). The structure-based design efforts targeting insect-specific C286 contributed to the successful development of selective AChE inhibitors specific to pests, without measurable inhibition of human AChE (Pang et al. 2012). Here, potential selective AChE inhibitors were screened from the ChemBridge database using DOCK 6 and AutoDock Vina. Lead-compound databases provide an extremely large number of lead-compound structure sources for screening (Qin et al. 2006). From the ChemBridge database, 39 potential selective AChE inhibitors were identified, and one of the 39 compounds (compound No. 8) was found to have an inhibitory effect comparable to that of eserine, but to be sparing of human AChE. No. 8 had dose-dependent toxicity against *T*. *urticae* but was still a bit lower than that of spirodiclofen. Structural differences between *Lucilia cuprina* and human AChE have also led to the identification of 19 previously unknown non-carbamate, non-organophosphate inhibitors, which are up to 335-fold more potent against the *L*. *cuprina* enzyme than against human AChE (Ilg et al. 2011). Aryl methylcarbamates bearing a β-branched 2-alkoxy or 2-thioalkyl group were found to possess good selectivity for inhibition of *A. gambiae* AChE over human AChE (Hartsel et al. 2012). Three selective AChE inhibitors specific for mites and less potent against human AChE were screened from the SPECS chemical lead-compound database (Bu et al. 2015b). Determining the complex structures of insecticide-bound AChE not only provides insight into how insecticides work but also helps to design novel selective insecticides through the systematic comparison of the structures from targeted and nontargeted species.

In the pot experiment, higher concentration of compound No. 8 was needed to get similar control effects on *T. urticae* as that of spirodiclofen, and its effects were concentration related (Fig. 2). Moreover, spraying of compound No. 8 had no effects on the growth of strawberry (Fig. 3). The toxicity evaluation suggested that compound No. 8 had no significant effects on honey bee and *N. californicus*. Thus, compound No. 8 is safe for honey bees and *N. californicus* in our experiments (Table 4). The compound No. 8 has piperidine moiety. Piperidine derivatives were displayed to be potent insecticides against the *Aedes aegypti* mosquitoes (Lima et al. 2022). Pyrethroids have been developed as insecticides by modification of pyrethrin structure, and they tend to be more effective than natural pyrethrins while they are less toxic to mammals (Ujihara 2019). Osthole-based isooxazoline derivatives showed better control efficacy against *P. xylostella* and relatively low toxicity toward nontarget organism than rotenone (Liu et al. 2022). Further work is required to optimize the potency of the AChE inhibitor that we have obtained (compound No. 8). Structure-based virtual screening, compared with biological and chemical screening, as a high-throughput screening tool, facilitates the development of new pesticides (Qin et al. 2006). The novel, selective AChE inhibitor with a unique structure can be used as a lead compound for developing pesticides with no effect on human beings. We are now working to identify more potent derivatives.

Restrictions on the use of some pesticides in Europe due to their potential adverse effects on bees have highlighted the need for future pesticides to have the ability to control pests while sparing bees (Pang et al. 2012). Although such requirements are difficult and complex, they could be adequately addressed by the innovations that continue to be brought to many fields, such as structural genomics, commodity cluster computing, and interdisciplinary research technologies (Pang et al. 2012). Selective AChE inhibitors hold great promise as effective and environmentally safe pesticides, particularly in light of the new requirement that new pesticides must spare honey bees and predatory mites.

## Supplementary Material

iead073_suppl_Supplementary_Table_S1

iead073_suppl_Supplementary_Table_S2

## References

[CIT0001] Adesanya AW , Cardenas A, Lavine MD, Walsh DB, Lavine LC, Zhu F. RNA interference of NADPH-cytochrome P450 reductase increases susceptibilities to multiple acaricides in *Tetranychus urticae*. Pestic Biochem Physiol. 2020:165:104550. 10.1016/j.pestbp.2020.02.016 32359548

[CIT0002] Allen WJ , Balius Te Fau - Mukherjee S, Mukherjee S, Fau - Brozell SR, Brozell Sr Fau - Moustakas DT, Moustakas Dt Fau - Lang PT, Lang Pt Fau - Case DA, Case Da Fau - Kuntz ID, Kuntz Id Fau - Rizzo RC, Rizzo RC. DOCK 6: impact of new features and current docking performance. J Comput Chem. 2015:36(15):1132–1156. 25914306 10.1002/jcc.23905 PMC4469538

[CIT0003] Anazawa Y , Tomita T, Fau - Aiki Y, Aiki Y, Fau - Kozaki T, Kozaki T, Fau - Kono Y, Kono Y. Sequence of a cDNA encoding acetylcholinesterase from susceptible and resistant two-spotted spider mite, *Tetranychus urticae*. Insect Biochem Mol Biol. 2003:33(5):509–514. 12706630 10.1016/s0965-1748(03)00025-0

[CIT0004] Bu C , Peng B, Cao Y, Wang X, Chen Q, Li J, Shi G. Novel and selective acetylcholinesterase inhibitors for *Tetranychus cinnabarinus* (Acari: Tetranychidae). Insect Biochem Mol Biol. 2015b:66:129–135. 10.1016/j.ibmb.2015.10.012 26520174

[CIT0005] Bu C-Y , Feng X-J, Wang X-Q, Cao Y, Wang Y-N, Chen Q, Gao P, Peng B, Li J-L, Han J-Y, et al. Cloning and characterization of the acetylcholinesterase1 gene of *Tetranychus cinnabarinus* (Acari: Tetranychidae). J Econ Entomol. 2015a:108(2):769–779. 10.1093/jee/tou046 26470189

[CIT0006] Cao Y , Li L. Improved protein-ligand binding affinity prediction by using a curvature-dependent surface-area model. Bioinformatics. 2014:30(12):1674–1680. 10.1093/bioinformatics/btu104 24563257

[CIT0007] Cao Y , Song L, Fau - Miao Z, Miao Z, Fau - Hu Y, Hu Y, Fau - Tian L, Tian L, Fau - Jiang T, Jiang T. Improved side-chain modeling by coupling clash-detection guided iterative search with rotamer relaxation. Bioinformatics. 2011:27(6):785–790. 21216772 10.1093/bioinformatics/btr009

[CIT0008] Dai PA-OX , Jack CJ, Mortensen AN, Ellis JD. Acute toxicity of five pesticides to *Apis mellifera* larvae reared in vitro. Pest Manag Sci. 2017:73(11):2282–2286. 10.1002/ps.4608 28485079

[CIT0009] Dai PA-OX , Yan Z, Ma S, Yang Y, Wang Q, Hou C, Wu Y, Liu Y, Diao Q. The herbicide glyphosate negatively affects midgut bacterial communities and survival of honey bee during larvae reared in vitro. J Agric Food Chem. 2018:66(29):7786–7793. 10.1021/acs.jafc.8b02212 29992812

[CIT0010] Dermauw W , Jonckheere W, Riga M, Livadaras I, Vontas J, Van Leeuwen T. Targeted mutagenesis using CRISPR-Cas9 in the chelicerate herbivore *Tetranychus urticae*. Insect Biochem Mol Biol. 2020:120:103347. 10.1016/j.ibmb.2020.103347 32114158

[CIT0011] El-Bialy SM , El-Mahrouk ME, Elesawy T, Omara AA-O, Elbehiry FA-O, El-Ramady HA-OX, Áron BA-O, Prokisch J, Brevik EA-O, Solberg SØ A-O. Biological nanofertilizers to enhance growth potential of strawberry seedlings by boosting photosynthetic pigments, plant enzymatic antioxidants, and nutritional status. Plants (Basel). 2023:12(2):302. 10.3390/plants12020302 36679014 PMC9865313

[CIT0012] Grbić M , Van Leeuwen T, Clark RM, Rombauts S, Rouzé P, Grbić V, Osborne EJ, Dermauw W, Ngoc PC, Ortego F, et al. The genome of *Tetranychus urticae* reveals herbivorous pest adaptations. Nature. 2011:479(7374):487–492. 10.1038/nature10640 22113690 PMC4856440

[CIT0013] Hadiatullah HA-OX , Zhang Y, Samurkas A, Xie Y, Sundarraj R, Zuilhof H, Qiao J, Yuchi ZA-O. Recent progress in the structural study of ion channels as insecticide targets. Insect Sci. 2022:29(6):1522–1551. 10.1111/1744-7917.13032 35575601

[CIT0014] Hartsel JA , Wong Dm Fau - Mutunga JM, Mutunga Jm Fau - Ma M, Ma M, Fau - Anderson TD, Anderson Td Fau - Wysinski A, Wysinski A, Fau - Islam R, Islam R, Fau - Wong EA, et al. Re-engineering aryl methylcarbamates to confer high selectivity for inhibition of *Anopheles gambiae* versus human acetylcholinesterase. Bioorg Med Chem Lett. 2012:22(14):4593–4598. 22738634 10.1016/j.bmcl.2012.05.103 PMC3389130

[CIT0015] Ilg T , Cramer J, Fau - Lutz J, Lutz J, Fau - Noack S, Noack S, Fau - Schmitt H, Schmitt H, Fau - Williams H, Williams H, et al. The characterization of *Lucilia cuprina* acetylcholinesterase as a drug target, and the identification of novel inhibitors by high throughput screening. Insect Biochem Mol Biol. 2011:41(7):470–483. 21530657 10.1016/j.ibmb.2011.04.003

[CIT0016] Kwon DH , Choi Jy Fau - Je YH, Je Yh Fau - Lee SH, Lee SH. The overexpression of acetylcholinesterase compensates for the reduced catalytic activity caused by resistance-conferring mutations in *Tetranychus urticae*. Insect Biochem Mol Biol. 2012:42(3):212–219. 22198354 10.1016/j.ibmb.2011.12.003

[CIT0017] Lang GJ , Zhu Ky Fau - Zhang C-X, Zhang CX. Can acetylcholinesterase serve as a target for developing more selective insecticides? Curr Drug Targets. 2012:13:495–501. 22280346 10.2174/138945012799499712

[CIT0018] Li C , Cao Y, Yang J, Li M, Li B, Bu CA-O. Acetylcholinesterase target sites for developing environmentally friendly insecticides against *Tetranychus urticae* (Acari: Tetranychidae). Exp Appl Acarol. 2021:84(2):419–431. 10.1007/s10493-021-00624-4 33914192

[CIT0019] Lima LR , Bastos RS, Ferreira EFB, Leão RP, Araújo PHF, Pita SSDR, De Freitas HF, Espejo-Román JM, Dos Santos ELVS, Ramos RDS, et al. Identification of potential new *Aedes aegypti* juvenile hormone inhibitors from N-acyl piperidine derivatives: a bioinformatics approach. Int J Mol Sci. 2022:23(17):9927. 10.3390/ijms23179927 36077329 PMC9456062

[CIT0020] Liu Z , Han M, Yan X, Cheng W, Tang Z, Cui L, Yang R, Guo Y. Design, synthesis, and biological evaluation of novel osthole-based isoxazoline derivatives as insecticide candidates. J Agric Food Chem. 2022:70(26):7921–7928. 10.1021/acs.jafc.2c01925 35731949

[CIT0021] O’Boyle NM , Banck M, Fau - James CA, James Ca Fau - Morley C, Morley C, Fau - Vandermeersch T, Vandermeersch T, Fau - Hutchison GR, Hutchison GR. Open Babel: an open chemical toolbox. J Cheminform. 2011:3:33. 21982300 10.1186/1758-2946-3-33 PMC3198950

[CIT0022] Ochiai N , Mizuno M, Fau - Mimori N, Mimori N, Fau - Miyake T, Miyake T, Fau - Dekeyser M, Dekeyser M, Fau - Canlas LJ, Canlas Lj Fau - Takeda M, et al. Toxicity of bifenazate and its principal active metabolite, diazene, to *Tetranychus urticae* and *Panonychus citri* and their relative toxicity to the predaceous mites, *Phytoseiulus persimilis* and *Neoseiulus californicus*. Exp Appl Acarol. 2007:43(3):181–197. 17972019 10.1007/s10493-007-9115-9

[CIT0023] Pang YP , Brimijoin S, Fau - Ragsdale DW, Ragsdale Dw Fau - Zhu KY, Zhu Ky Fau - Suranyi R, Suranyi R. Novel and viable acetylcholinesterase target site for developing effective and environmentally safe insecticides. Curr Drug Targets. 2012:13(4):471–482. 22280344 10.2174/138945012799499703 PMC3343382

[CIT0024] Pang YP , Singh S, Fau - Gao Y, Gao Y, Fau - Lassiter TL, Lassiter T, Fau - Mishra RK, Mishra R, Fau - Zhu KY, Zhu Ky Fau - Brimijoin S, et al. Selective and irreversible inhibitors of aphid acetylcholinesterases: steps toward human-safe insecticides. PLoS One. 2009:4(2):e4349. 19194505 10.1371/journal.pone.0004349 PMC2632757

[CIT0025] Qin Z , Zhang J, Fau - Xu B, Xu B, Fau - Chen L, Chen L, Fau - Wu Y, Wu Y, Fau - Yang X, Yang X, et al. Structure-based discovery of inhibitors of the YycG histidine kinase: new chemical leads to combat *Staphylococcus epidermidis* infections. BMC Microbiol. 2006:6:96. 17094812 10.1186/1471-2180-6-96 PMC1660542

[CIT0026] Samaras KA-O , Mourtiadou S, Arampatzis TA-O, Kakagianni MA-O, Feka M, Wäckers F, Papadopoulou KA-O, Broufas GA-O, Pappas MA-O. Plant-mediated effects of beneficial microbes and a plant strengthener against spider mites in tomato. Plants (Basel). 2023:12(4):938. 10.3390/plants12040938 36840286 PMC9959994

[CIT0027] Trott O , Olson AJ. AutoDock Vina: improving the speed and accuracy of docking with a new scoring function, efficient optimization, and multithreading. J Comput Chem. 2010:31(2):455–461. 10.1002/jcc.21334 19499576 PMC3041641

[CIT0028] Ujihara K. The history of extensive structural modifications of pyrethroids. J Pestic Sci. 2019:44(4):215–224. 10.1584/jpestics.D19-102 31777441 PMC6861428

[CIT0029] Van Leeuwen T , Dermauw W. The molecular evolution of xenobiotic metabolism and resistance in chelicerate mites. Annu Rev Entomol. 2016:61:475–498. 10.1146/annurev-ento-010715-023907 26982444

[CIT0030] Yang J , Zhang Y. I-TASSER server: new development for protein structure and function predictions. Nucleic Acids Res. 2015:43(W1):W174–W181. 10.1093/nar/gkv342 25883148 PMC4489253

[CIT0031] Zhang Y. I-TASSER server for protein 3D structure prediction. BMC Bioinf. 2008:9:40. 10.1186/1471-2105-9-40 PMC2245901 18215316

[CIT0032] Zheng W , Zhang C, Wuyun Q, Pearce R, Li Y, Zhang Y. LOMETS2: improved meta-threading server for fold-recognition and structure-based function annotation for distant-homology proteins. Nucleic Acids Res. 2019:47(W1):W429–W436. 10.1093/nar/gkz384 31081035 PMC6602514

