# Supplementary material for: Discovery selective acetylcholinesterase inhibitors to control Tetranychus urticae (Acari: Tetranychidae)
Source: J Insect Sci. 2023 Aug 14;23(4):19. doi: 10.1093/jisesa/iead073 (PMC10424716; doi:10.1093/jisesa/iead073)
Supplement: iead073_suppl_Supplementary_Table_S1 [file iead073_suppl_supplementary_table_s1.docx]

**Table S1** The compounds obtained by virtual screening through ChemBridge

| Code | ChemBridge ID | Chemical name |
| --- | --- | --- |
| 1 | 72009069 | cis-3-[(S*)-(2,3-dihydro-1H-inden-2-ylamino)(pyridin-2-yl)methyl]cyclobutanol |
| 2 | 93971100 | N-methyl-1-(4-methylpyridin-2-yl)-N-(2-phenylethyl)propan-2-amine |
| 3 | 25738362 | 3-(1H-indol-3-yl)-N-[2-(4-methyl-5,6,7,8-tetrahydroquinazolin-2-yl)ethyl]propanamide |
| 4 | 85224744 | 2-({methyl[1-methyl-2-(3-methylpyridin-2-yl)ethyl]amino}methyl)benzoic acid |
| 5 | 67130753 | 3-{2-[2-(4-fluorophenyl)azetidin-1-yl]ethyl}-1,3-benzoxazol-2(3H)-one |
| 6 | 57289427 | N-methyl-N-[1-methyl-2-(3-methylpyridin-2-yl)ethyl]cyclopentanecarboxamide |
| 7 | 93511284 | 2-(1H-indol-3-yl)-N-methyl-N-[1-methyl-2-(4-methylpyridin-2-yl)ethyl]acetamide |
| 8 | 86520749 | 2-(2,3-dihydro-1H-inden-1-yl)-N-[2-(3-pyridinyl)ethyl]acetamide |
| 9 | 22216181 | 1-(1-benzofuran-5-ylcarbonyl)-4-(benzyloxy)piperidine |
| 10 | 94778243 | 1-(1-benzofuran-5-ylmethyl)-4-(3-fluorobenzyl)-1,4-diazepane |
| 11 | 63822868 | [9-(1-benzofuran-5-ylmethyl)-3-oxo-2,9-diazaspiro[5.5]undec-2-yl]acetic acid |
| 12 | 23322618 | 3-{2-oxo-2-[(1R*,5S*)-7-oxo-3,6-diazabicyclo[3.2.2]non-3-yl]ethyl}-1,3-benzoxazol-2(3H)-one |
| 13 | 58907370 | 3-[2-(2-pyridin-3-ylmorpholin-4-yl)ethyl]-1,3-benzoxazol-2(3H)-one |
| 14 | 53866088 | N-[rac-(1S,5S,6S,7R)-7-(methoxymethyl)-2-oxabicyclo[3.2.0]hept-6-yl]-3-(5-methyl-2-oxo-1,3-benzoxazol-3(2H)-yl)propanamide |
| 15 | 77679919 | 3-{2-[3-(2-fluorophenyl)-1-pyrrolidinyl]-2-oxoethyl}-1,3-benzoxazol-2(3H)-one |
| 16 | 19112510 | 3-{2-[rac-(3aR,6R,7aS)-3a,6-dimethoxyoctahydro-1H-indol-1-yl]ethyl}-1,3-benzoxazol-2(3H)-one |
| 17 | 40064958 | N~1~-(3-chlorobenzyl)-N~3~-(3-hydroxybenzyl)-N~3~-methyl-beta-alaninamide |
| 18 | 54774802 | 3-(3-chlorophenoxy)-1-(3-methylbutanoyl)azetidine |
| 19 | 89819079 | N-methyl-1-pyridin-3-yl-N-(quinolin-2-ylmethyl)ethanamine |
| 20 | 17722140 | 4,4,4-trifluoro-N-[1-methyl-2-(3-methylpyridin-2-yl)ethyl]butanamide |
| 21 | 14249439 | 2-(2,3-dihydro-1H-inden-2-yl)-N-[1-(cis-3-hydroxycyclobutyl)-2-pyridin-2-ylethyl]acetamide |
| 22 | 88723037 | N-[3-(1H-1,2,3-triazol-1-yl)propyl]-2,3,4,9-tetrahydro-1H-carbazole-1-carboxamide |
| 23 | 92682634 | 2-{2-[1-(pyridin-3-ylacetyl)piperidin-2-yl]ethyl}pyridine |
| 24 | 14595504 | 2-(7-fluoro-2-methyl-1H-indol-3-yl)-N-[4-(2-pyridinyl)butyl]acetamide |
| 25 | 75029547 | 9-[(3-methyl-1-benzofuran-2-yl)methyl]-3-oxa-9-azaspiro[5.5]undecane |
| 26 | 71689782 | 4-(isoquinolin-4-ylmethyl)-1-(2-methoxyethyl)pyrrolidin-2-one |
| 27 | 94145715 | 1-(3-furylmethyl)-4-(2-phenoxyethyl)piperazine |
| 28 | 94788265 | N-(3-methoxybenzyl)-N-(2-methoxy-1-methylethyl)-1-benzofuran-3-carboxamide |
| 29 | 54639423 | 2,4-dichloro-6-(2,8-diazaspiro[4.5]dec-2-ylmethyl)phenol |
| 30 | 10162960 | 3-chloro-N-ethyl-4-hydroxy-N-(2-pyrrolidin-1-ylethyl)benzamide |
| 31 | 28664114 | 2-(3-chloro-4-hydroxyphenyl)-N-{[trans-4-(hydroxymethyl)cyclohexyl]methyl}acetamide |
| 32 | 18161734 | (8-syn)-3-(2,6-dichloro-3-hydroxybenzyl)-3-azabicyclo[3.2.1]octan-8-ol |
| 33 | 55978695 | 2-(2,4-dichlorophenoxy)-N-[2-(4-pyridinyl)ethyl]acetamide |
| 34 | 24161669 | 2-(2,4-dichlorophenoxy)-N-(tetrahydro-2H-pyran-4-yl)acetamide |
| 35 | 39379171 | 3-{4-oxo-4-[2-(2-pyridinyl)-1-pyrrolidinyl]butyl}-1H-indole |
| 36 | 93121808 | 1-(2-methoxyethyl)-4-(5,6,7,8-tetrahydroquinolin-2-ylmethyl)pyrrolidin-2-one |
| Code | ChemBridge ID | Chemical name |
| 37 | 70552910 | N-(4-pyridin-2-ylbutyl)-3-azaspiro[5.5]undecan-9-amine |
| 38 | 69603305 | pyridin-2-yl[1-(3-pyridin-3-ylpropanoyl)piperidin-4-yl]methanol |
| 39 | 54010160 | 2-(1H-indol-3-yl)-N-[2-(4-methyl-5,6,7,8-tetrahydroquinazolin-2-yl)ethyl]acetamide |
