# Supplementary material for: Discovery selective acetylcholinesterase inhibitors to control Tetranychus urticae (Acari: Tetranychidae)
Source: J Insect Sci. 2023 Aug 14;23(4):19. doi: 10.1093/jisesa/iead073 (PMC10424716; doi:10.1093/jisesa/iead073)
Supplement: iead073_suppl_Supplementary_Table_S2 [file iead073_suppl_supplementary_table_s2.docx]

**Table S2** The field layout of strawberry seedlings at the trifoliate stage

| Numbers of mites inoculated on each seedlings | Compound No. 8 (mg/mL) | | | | spirodiclofen (mg/mL) | | | |
| --- | --- | --- | --- | --- | --- | --- | --- | --- |
|  | 0 | 1 | 3 | 5 | 0 | 0.06 | 0.12 | 0.24 |
| 0 | 4 seedlings | 4 seedlings | 4 seedlings | 4 seedlings | 4 seedlings | 4 seedlings | 4 seedlings | 4 seedlings |
| 15 | 4 seedlings | 4 seedlings | 4 seedlings | 4 seedlings | 4 seedlings | 4 seedlings | 4 seedlings | 4 seedlings |
| 30 | 4 seedlings | 4 seedlings | 4 seedlings | 4 seedlings | 4 seedlings | 4 seedlings | 4 seedlings | 4 seedlings |
